# Facial Scans in Clinical Dentistry and Related Research: A Scoping Review

**DOI:** 10.7759/cureus.81662

**Published:** 2025-04-03

**Authors:** Takahiro Shuto, Yuichi Mine, Akina Tani, Tsuyoshi Taji, Takeshi Murayama

**Affiliations:** 1 Department of Oral Health Engineering, Faculty of Health Sciences, Osaka Dental University, Osaka, JPN; 2 Project Research Center for Integrating Digital Dentistry, Hiroshima University, Hiroshima, JPN; 3 Department of Medical Systems Engineering, Graduate School of Biomedical and Health Sciences, Hiroshima University, Hiroshima, JPN; 4 Department of Oral Health Sciences, Faculty of Health Sciences, Osaka Dental University, Osaka, JPN; 5 Department of Oral Biology and Engineering, Graduate School of Biomedical and Health Sciences, Hiroshima University, Hiroshima, JPN

**Keywords:** clinical application, clinical dentistry, facial scan, scanning technologies, scoping review

## Abstract

This scoping review examined the adoption of three-dimensional (3D) facial scanning technology in clinical dentistry, focusing on the range of scanning techniques, clinical applications, and related implementation trends. A systematic literature search of three major databases identified studies published between January 2020 and June 2024 that described 3D facial scanning of human participants in clinical dental settings. After screening 224 records, 48 from 19 countries met the inclusion criteria. Both clinical studies and case/technical reports showed that facial scanning was used in multiple specialties, including orthodontics, prosthodontics, and maxillofacial surgery, primarily for diagnosis, treatment planning, and outcome assessment. The technologies such as stereophotogrammetry, structured light scanning, laser scanning, and mobile device-based solutions vary in accuracy and ease of integration. Mobile scanning, in particular, is growing in importance due to lower cost, accessibility, and compatibility with digital workflows. Despite these advances, standardized protocols for integrating facial scans with other digital records, such as cone beam computed tomography and intraoral scans, remain underdeveloped. This review demonstrates the growing importance of 3D facial scanning in improving clinician-patient communication and identifies areas where further research is needed, including long-term validation, cost-effectiveness, and standardized data management.

## Introduction and background

In recent years, three-dimensional (3D) facial scanning technology has become a transformative tool in clinical dentistry, offering new possibilities for diagnosis, treatment planning and outcome evaluation [1]. The integration of facial scanning into the dental practice represents a significant advance over traditional 2D photography and manual measurements [2], providing clinicians with accurate digital representations of patients' facial features and their relationship to dental structures [1].

The adoption of facial scanning in clinical dentistry has been driven by several factors. First, the technology allows for objective and quantitative assessment of facial morphology, which is crucial for various dental procedures, particularly in specialties such as orthodontics, prosthodontics, and maxillofacial surgery [3-6]. Second, the ability to create detailed 3D models enables better communication with patients and more precise treatment planning [7,8]. Third, recent technological advances have made facial scanning more accessible, with the introduction of portable devices and improved software integration capabilities [9,10].

However, despite its growing clinical promise, several limitations continue to prevent the widespread adoption of 3D facial scanning in dentistry. High cost remains a major barrier, as professional-grade stereophotogrammetry or structured light scanning systems often require substantial investments in hardware, proprietary software, and maintenance [9,10]. Accessibility is further limited in certain regions or smaller clinics that lack the infrastructure to support digital imaging workflows [10]. In addition, the lack of standardized scanning protocols, including variations in patient positioning, landmark selection, and data registration, results in heterogeneous methodologies across studies and practices [9]. These inconsistencies complicate efforts to compare results or implement consistent workflows. 

Various facial scanning technologies are currently available, including stereophotogrammetry [11], 3D laser scanning [12], structured light scanning [13], and solutions integrated with cone-beam computed tomography (CBCT) [6]. Each of these technologies offers different advantages in terms of accuracy, speed, cost, and ease of use. There have been numerous reviews of facial scanning in dentistry [1-3,10,14]; most have focused primarily on scanning accuracy and technical validation. Understanding these practical applications is critical for several reasons: it can help identify emerging clinical workflows, guide technology selection based on real-world usage patterns, and highlight areas where further clinical validation is needed.

Therefore, this scoping review aims to address two fundamental questions: 1) What are the purposes for which facial scanning has been researched and used clinically in dentistry? and 2) What facial scanning techniques are being researched and used in clinical practice? By systematically examining the literature from January 2020 to June 2024, this review aims to provide a comprehensive overview of the current state of facial scanning in clinical dentistry, including both research applications and clinical implementations. The results of this review will be particularly relevant to dental practitioners considering the adoption of facial scanning technology, researchers planning future studies, and technology developers working on next-generation scanning solutions. 

## Review

Materials and methods

Protocol and Registration

This scoping review was conducted following the Preferred Reporting Items for Systematic reviews and Meta-Analyses extension for Scoping Reviews (PRISMA-ScR) guidelines [15]. The study protocol was registered on the Open Science Framework (https://osf.io/rjw7e/, DOI 10.17605/OSF.IO/RJW7E) after the initial screening stage.

Search Strategy

A comprehensive electronic literature search was performed using three major databases: PubMed/MEDLINE, Scopus, and Web of Science. The search period was set from January 1, 2020, to June 1, 2024. The search terms combined concepts related to facial scanning, dentistry, and clinical applications. The specific search strategy implemented across all databases was: ("Facial scan" OR "Facial scanner" OR "3D facial model" OR "facial imaging") AND (dentistry OR dental) AND (diagnosis OR "treatment planning" OR "treatment evaluation" OR simulation OR "clinical application" OR "clinical use").

Eligibility Criteria

This study included peer-reviewed journal articles published in English that focused on facial scanning techniques in clinical dentistry. Studies were considered eligible if they involved human participants and/or patients and reported on the use of facial scanning techniques such as stereophotogrammetry, 3D laser scanning, structured light scanning, intra-oral scanning, or cone-beam computed tomography. We excluded articles without abstracts, review articles, editorials, and studies conducting in vitro experiments using plaster models or mannequin heads. Conference abstracts and proceedings were also excluded from the review.

Study Selection

The study selection process was conducted in multiple stages. Initially, all identified articles were screened for duplicates by one reviewer (YM). Following duplicate removal, two independent reviewers (TS, YM and AT) assessed the titles and abstracts of the remaining articles against the predefined eligibility criteria. The full texts of potentially eligible articles were then retrieved and evaluated by the same three reviewers. Any disagreements between reviewers during the selection process were resolved through consensus after a thorough review of the full text. In cases where consensus could not be reached, a senior researcher (TT) made the final decision.

Data Extraction and Analysis

For clinical studies, we extracted information regarding author details, publication year, country of the first author, research specialty, study objectives, patient demographics, facial scanning devices, processing software used, and clinical outcomes. For case reports and technical reports, we collected data on author information, publication year, country of origin, specialty, study objectives, technical specifications, patient case details, scanning devices, processing software, and clinical applications. This distinction in data extraction allowed for a comprehensive understanding of both systematic research approaches and individual case implementations.

Data Synthesis

To visualize the geographical distribution of the included studies, a choropleth map was created using Python (version 3.6.4) with the Plotly library. This visualization helped to identify geographic patterns in research activity related to facial scanning in clinical dentistry. The synthesis of findings focused on identifying patterns in facial scanning applications, technological trends, clinical outcomes, and methodological approaches. All findings were synthesized descriptively to provide a comprehensive overview of the current state of facial scanning in clinical dentistry.

Results

Study Selection and Characteristics

**Figure 1:**
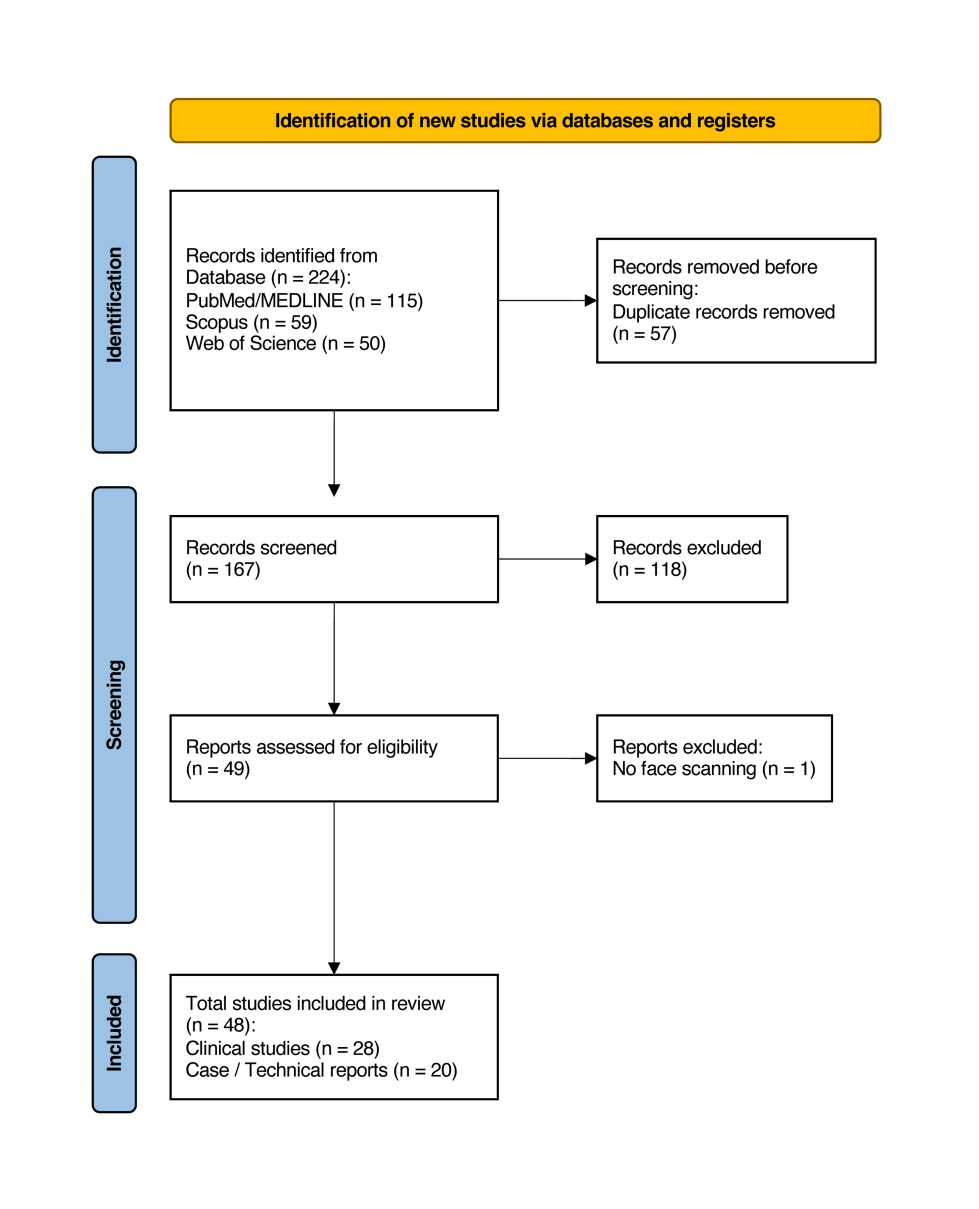
Flowchart illustrating the screening process used to select the papers included in this study The flowchart was adapted from the Preferred Reporting Items for Systematic reviews and Meta-Analyses (PRISMA) guidelines to meet the criteria of a scoping review.

The search process was adapted to the requirements of a scoping review from the PRISMA flowchart [16]. A total of 224 records were identified through database searches, with PubMed/MEDLINE providing 115 records, Scopus 59 records, and Web of Science 50 records. After removal of 57 duplicates, 167 articles were screened based on title and abstract. After applying the inclusion and exclusion criteria, 48 articles were included in the final analysis (Figure 1) [17-64].

**Figure 2:**
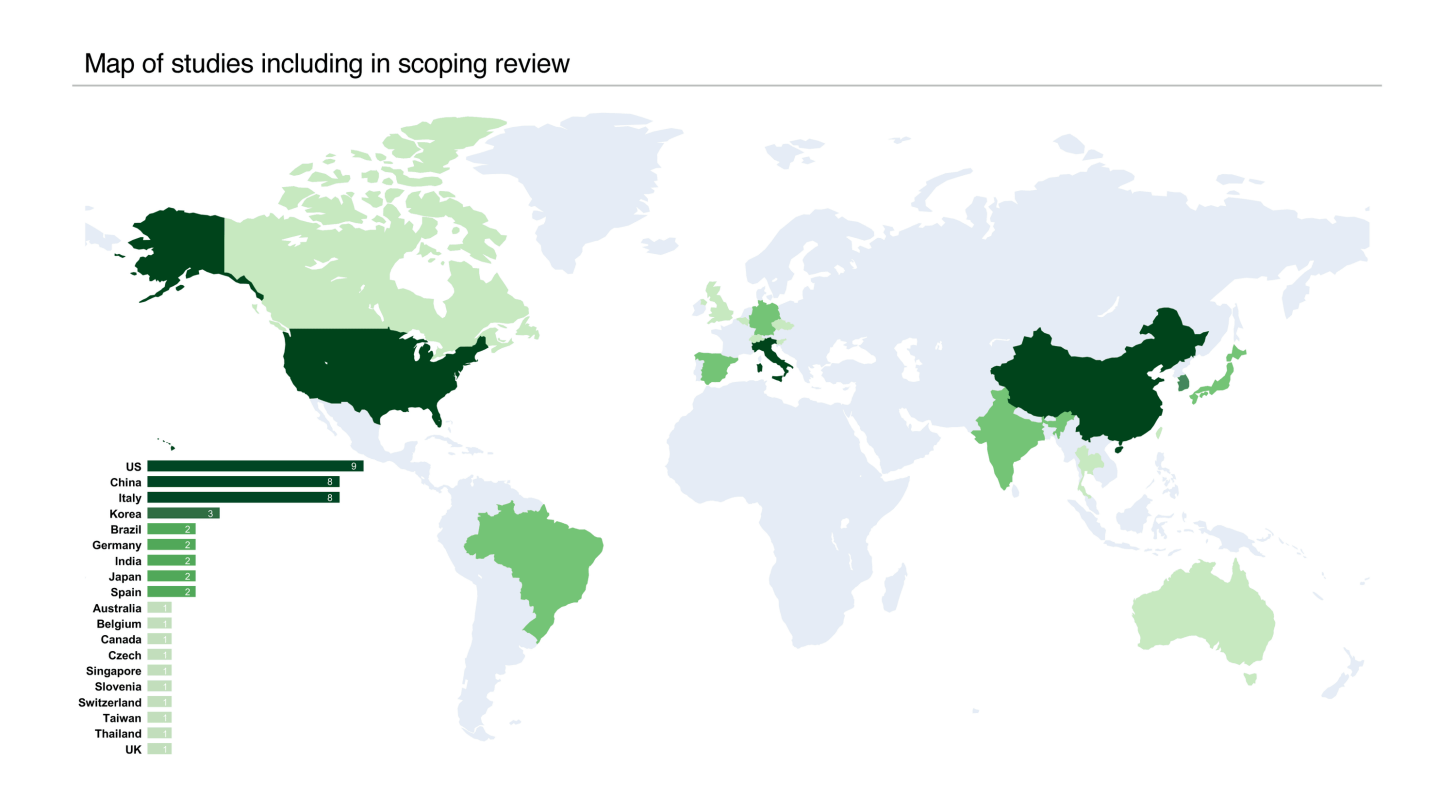
Map depicting the geographic distribution of studies included in the scoping review Darker shades represent a higher number of studies from each country, as detailed in the bar chart on the left.

As shown in Figure 2, these studies originated from 19 different countries, with the United States contributing nine studies, while China and Italy each contributed eight studies. The geographic distribution indicates a global interest in facial scanning technology, with particularly strong research activity in North America, East Asia, and Europe.

Temporal Distribution and Research Fields

Analysis of the publication timeline from Tables 1, 2 shows an increasing trend in research output from 2020 to 2024. The field showed significant diversity in research specialties, with studies spanning multiple dental disciplines. Clinical studies (Table 1) were dominated by multidisciplinary research, followed by combined orthodontic and oral surgery studies. Case and technical reports (Table 2) showed a particular focus on prosthodontics and the implementation of digital workflows, especially in areas such as digital smile design and virtual treatment planning. The research objectives documented in both tables varied widely, from diagnostic applications and treatment planning to outcome evaluation, reflecting the versatility of facial scanning technology in dental practice. These were primarily postoperative assessments of tissue morphology, amount of change, and scanner accuracy. This parallel development of clinical research and technical implementation suggests a mature field where research findings are rapidly translated into clinical applications.

**Table 1:** Characteristics and summary of selected studies Abbreviations: FAS, fetal alcohol syndrome; 3D, three-dimensional; WBS, Williams-Beuren syndrome; CBCT, cone-bean computed tomography; BSSRO, bilateral sagittal split ramus osteotomy; PWPA, power function weighted Procrustes analysis; SARME, symmetry reference plane

Author	Year	Country	Specialty	Objective(s)
Blanck-Lubarsch et al. [17]	2020	Germany	Pediatric dentistry Orthodontics	The purpose of this study is to evaluate palatal depth and vertical facial measurements in children with FAS by using 3D scanning technology, and to offer additional objective diagnostic parameters that can aid in the early detection of FAS.
Piedra-Cascón et al. [18]	2020	Spain	Multidisciplinary	The purpose of this study was to assess the accuracy (including trueness and precision) of extraoral 3D facial reconstructions captured with a dual-structured light facial scanner, as well as to evaluate the interexaminer variability.
Revilla-León et al. [19]	2020a	US	Esthetic dentistry Prosthodontics	The purpose of this study was to examine how laypersons, dental students, and dentists perceive discrepancies in the maxillary dental midline and the occlusal plane when they evaluate their own 2D or 3D clinical simulations.
Revilla-León et al. [20]	2020b	US	Esthetic dentistry Prosthodontics	The purpose of this study was to examine how laypersons, dental students, and dentists perceive maxillary midline and occlusal plane discrepancies when evaluating 2D and 3D clinical simulations.
Awuti et al. [21]	2021	Japan	Prosthodontics	The purpose of this study was to geometrically evaluate the effect of a mandibular prosthesis on facial asymmetry in patients with two different types of mandibulectomy defects. In addition, it aimed to quantify the improvement in facial symmetry through prosthetic rehabilitation using 3D scanning technology.
Danneels et al. [22]	2021	Belgium	Orthodontics Paediatric dentistry	The purpose of this study was to determine whether 3D facial scans can detect WBS by objectively analyzing craniofacial, skeletal, and dental characteristics and comparing these findings to those of an unaffected control group.
Denadai et al. [23]	2021	Taiwan	Orthodontics Oral surgery	The purpose of this study was to evaluate the effects of simultaneous orthognathic surgery and cheek-specific fat grafting on facial appearance and midface volumetric and positional changes compared to isolated orthognathic surgery in adult women with a Class III skeletal deformity.
Friscia et al. [24]	2021	Italy	Orthodontics Oral surgery	The purpose of this study was to evaluate the efficacy of the Hilotherapy face mask in reducing facial edema following orthognathic surgery by examining facial surfaces using an innovative iPhone-based 3D facial scanning system.
Horn et al. [25]	2021	Switzerland	Multidisciplinary	The purpose of this study was to determine the dimensional characteristics of an attractive smile using self-assessment tools and three-dimensional surface imaging, specifically testing the hypothesis that smile width and smile height affect self-perceived smile attractiveness.
Pellitteri et al. [26]	2021	Italy	Multidisciplinary	The purpose of this study was to compare the accuracy of the Face Hunter facial scanner and the Dental Pro application with both manual measurements and each other, and to evaluate the reproducibility and reliability of these digital scanning systems for facial surface capture.
Blanck-Lubarsch et al. [27]	2022	Germany	Pediatric dentistry Orthodontics	The purpose of this study was to use existing 3D metric facial data of FAS patients to identify machine learning methods that could improve and objectify the diagnostic process for FAS. Three methods were compared: decision trees, support vector machines, and k-nearest neighbors.
Eliasova et al. [28]	2022	Czech	Multidisciplinary	The purpose of this study was to compare the accuracy of 3D facial images acquired using the 3dMD facial scanner and a CBCT unit, and to characterize changes in facial shape caused by maxillofacial surgery between the preoperative and postoperative states.
Lee et al. [29]	2022	Singapore	Orthodontics Oral surgery	The purpose of this study was to determine the validity of ProPlan CMF software in predicting soft tissue morphology outcomes after bimaxillary orthognathic surgery in Chinese patients with skeletal Class III.
Raffone et al. [30]	2022	Italy	Multidisciplinary	The purpose of this study was to investigate the trueness and precision of a low-cost, portable face scanner using two different scanning techniques: the “Free Technique,” in which patients rotate their heads, and the “Slider Technique,” in which the scanner moves around a fixed head position.
Revilla-León et al. [31]	2022a	US	Esthetic dentistry Prosthodontics	The purpose of this study was to compare self-perception ratings and self-representation preferences between 2D and 3D facial reconstructions among laypersons, dental students, and dentists, and to evaluate how each group perceives and prefers these visualization methods for treatment planning.
Revilla-León et al. [32]	2022b	US	Prosthodontics	The purpose of this study was to determine and compare the accuracy (trueness and precision) of a virtual patient generated by the superimposition of facial and intraoral digital scans, guided by two different scanning body systems.
Xu et al. [33]	2022	China	Orthodontics Oral surgery	The purpose of this study was to determine and compare the accuracy (trueness and precision) of a virtual patient generated by the superimposition of facial and intraoral digital scans, guided by two different scanning body systems.
Zhu et al. [34]	2022	China	Orthodontics Oral surgery	The purpose of this study was to investigate and evaluate a novel mathematical algorithm based on PWPA for determining 3D facial asymmetry in patients with mandibular deviation.
Zupan et al. [35]	2022	Slovenia	Orthodontics Oral surgery	The purpose of this study was to evaluate three-dimensional facial soft tissue changes following SARME by analyzing preoperative and postoperative 3D facial scans, in order to understand both transverse and sagittal changes in facial morphology.
Aljawad et al. [36]	2023	Korea	Multidisciplinary	The purpose of this study was to evaluate the accuracy of 3D facial scans acquired with a low-cost facial scanner, compared to those acquired via CBCT, by measuring and comparing anthropometric measurements between the two imaging modalities.
Andrews et al. [37]	2023	Canada	Multidisciplinary	The purpose of this study was to validate both the trueness and precision of the iPhone 11 Pro smartphone TrueDepth NIR scanner with the Bellus3D Face app in capturing 3D facial images, compared to the conventional 3dMDface stereophotogrammetry system.
Cascos et al. [38]	2023	Spain	Multidisciplinary	The purpose of this study was to evaluate the accuracy and utility of a dual-structure light 3D facial scanner compared to 2D photography for facial analysis at maximum intercuspation and smile positions. The reliability of both methods was assessed using manual measurements as the gold standard.
Hou et al. [39]	2023	China	Orthodontics Oral surgery	The purpose of this study was to evaluate the accuracy of soft tissue prediction in patients undergoing orthognathic surgery to correct skeletal Class III malocclusions using maxillofacial regional aesthetic units.
Nogueira et al. [40]	2023	Brazil	Multidisciplinary	The purpose of this study was to evaluate the accuracy of facial measurements on three-dimensional images obtained using a new photogrammetric scanner (Cloner 3D) by comparing digital measurements with manual caliper measurements, and to assess inter- and intra-examiner reliability
Doan et al. [41]	2024	Australia	Multidisciplinary	The purpose of this study was to evaluate the accuracy of the Bellus3D ARC-7 camera by comparing measurements of soft tissue facial landmarks between digital scans and manual caliper measurements, and to assess the intra- and inter-examiner reliability of both methods.
Ercal et al. [42]	2024	UK	Implantology	The purpose of this study was to evaluate 3D morphometric facial changes at different follow-ups after single implant placement using a facial scanner, and to describe the challenges in image capturing/analysis when using facial scanners to assess 3D soft tissue changes.
Farronato et al. [43]	2024	Italy	Orthodontics	The purpose of this study was to evaluate the differences between CBCT-derived and facial scanner soft tissue representations, and to establish correlations between hard and soft tissue cephalometric measurements in healthy adults with normal skeletal relationships.
Jearanai et al. [44]	2024	Thailand	Orthodontics Oral surgery	The purpose of this study was to investigate the relationships between bilateral landmarks in the vertical dimension of facial asymmetry among patients with skeletal Class II and Class III malocclusions using 3D virtual head models created from CBCT and facial scans.

**Table 2:** Characteristics and summary of selected case and technical reports Abbreviations: 3D, three-dimensional; CBCT, cone-bean computed tomography; PMMA, polymethyl methacrylate; CAD/CAM, computer-aided design/computer-aided manufacturing

Author	Year	Country	Specialty	Objective(s)
Coachman et al. [45]	2020	Brazil	Prosthodontics	Introducing a new 3D digital smile design application for esthetic planning, smile simulation, chairside 3D virtual wax-up and trial restoration using portable devices that integrate 2D and 3D data for immediate chairside application.
Granata et al. [46]	2020	Italy	Prosthodontics	The objective was to describe a digital workflow for the superimposition of different 3D patient data files (intraoral scanner, CBCT, facial scanner) by a geometric occlusal registration prototype device.
Lo Russo et al. [47]	2020	Italy	Prosthodontics	To describe a technique for merging intraoral scans of edentulous arches with facial scans. The method integrates mobile phone facial scanning with intraoral scanning. This technique aims to optimize tooth placement in digital dentures.
Park et al. [48]	2020	US	Esthetic dentistry	To describe a digital workflow protocol for esthetic rehabilitation using direct composite restorations that combines facial digitization, intraoral scanning, and additive manufacturing technologies.
Li et al. [49]	2021	China	Esthetic dentistry	To describe the development and use of a virtual dental patient system with dynamic occlusion for crown lengthening surgery and esthetic restoration of maxillary anterior teeth.
Pérez-Giugovaz et al. [50]	2021	US	Prosthodontics	To describe a technique for obtaining a 3D virtual representation of a maxillary edentulous patient using an additively manufactured intraoral scan body for overdenture fabrication.
Sun et al. [51]	2021	China	Prosthodontics	The purpose of this technique was to present a digital protocol for integrating facial and intraoral scans to achieve predictable prosthetic design through digital facebow transfer and cross-articulation technique.
Alisha et al. [52]	2022	India	Orthodontics Pediatric dentistry	To develop and evaluate a novel metal frame device that enables stable and accurate 3D facial scanning of infants with cleft lip and palate, overcoming the challenges of head stabilization during scanning procedures.
Beretta et al. [53]	2022	Italy	Pediatric dentistry Orthodontics	To illustrate how new digital technologies (TrueDepth) can enable 3D integration of facial soft tissue scans and intraoral scans for orthodontic evaluation in children, providing a radiation-free diagnostic approach.
Campobasso et al. [54]	2022	Italy	Orthodontics	To provide an accurate, simple, and non-invasive method for matching an intraoral scan to a patient's facial scan without the need for CBCT, thereby reducing radiation exposure while maintaining diagnostic accuracy.
Gupta et al. [55]	2022	India	Oral surgery Prosthodontics	This case report describes a novel stereophotogrammetric method using Bellus 3D facial scanning software and rapid prototyping technology for PMMA cranioplast fabrication to rehabilitate a frontal cranial defect.
Park et al. [56]	2022	Korea	Esthetic dentistry	To present a methodology for creating a digital virtual patient by integrating CBCT, intraoral scan, and 3D facial scan with high accuracy using fiducial markers for precise registration.
Yang et al. [57]	2022	China	Prosthodontics	The purpose of this study is to propose a digital technique for registering intraoral scans to a virtual articulator and setting the patient-specific sagittal condylar inclination using the patient's cutaneous landmarks and a digital protrusive interocclusal record through a dental CAD software program.
Amin SA et al. [58]	2023	US	Prosthodontics	To describe a fully guided digital workflow using a 3D facial scanning approach for wax-up design, surgical implant planning, and fabrication of an interim full-arch implant-supported prosthesis after guided alveolar ridge reduction with fewer appointments.
Garaicoa et al. [59]	2023	US	Prosthodontics	To demonstrate a technique that integrates facial and dental scanners for treatment planning and execution of a tooth-borne full-mouth reconstruction with zirconia fixed prostheses, showing the protocol for combining facial and chairside CAD/CAM systems in a comprehensive rehabilitation case.
Wang et al. [60]	2023	China	Prosthodontics	To introduce a digital workflow for predicting facial aesthetics and optimizing prosthetic rehabilitation in patients with maxillofacial trauma using integrated CBCT and 3D facial scanning technologies.
Yang et al. [61]	2023	China	Prosthodontics	To reduce deformation during face scanning in the region between the lips' vermilion border and the teeth using a custom-made silicone matrix with blue screen technology for 3D digital smile design and implant planning.
Elbashti et al. [62]	2024	Japan	Prosthodontics	To present a simple protocol for registering intraoral scans to optical facial scans using nasal geometry as a matching reference, without the need for additional markers or complicated procedures, while maintaining clinical accuracy.
Lee et al. [63]	2024	Korea	Orthodontics	To present a method for the digital application of three-dimensional diagnosis and treatment using a virtual articulator and 3D data by integrating CBCT, intraoral scans, and facial scans.
Salloum [64]	2024	US	Prosthodontics	To introduce an innovative method for accurately registering facial and intraoral scans using a 3D-printed device that incorporates the nose as a stable reference point, enabling accurate merging of digital dental data for comprehensive treatment planning and improved patient communication.

Facial Scanning Technologies

The analysis of scanning technologies documented in Tables 3, 4 revealed two main categories of scanning systems: traditional professional systems and mobile device-based solutions. The professional systems documented in Table 3 included dedicated devices such as 3dMD systems (3dMD LLC, Atlanta, GA, USA), VECTRA H1 (Canfield Scientific, Parsippany, NJ, USA), and CBCT-integrated systems such as Planmeca ProMax 3D ProFace (Planmeca, Helsinki, Finland), which were primarily used in controlled clinical studies. Mobile solutions, in particular the Bellus3D system (Bellus3D Inc., Campbell, CA, USA), emerged as a frequently used technology in both clinical studies and case reports, implemented through various platforms such as iPhones, iPads and Android tablets. Intraoral scanners were also integrated and heavily used in case reports in Table 4.

**Table 3:** Data acquisition method and patient information of selected clinical studies Abbreviations: FAS, fetal alcohol syndrome; 3D, three-dimensional; WBS, Williams-Beuren syndrome; CBCT, cone-bean computed tomography; BSSRO, bilateral sagittal split ramus osteotomy; PWPA, power function weighted Procrustes analysis; SARME, symmetry reference plane

Author	Patient information	Age	Sex and sample size	Facial scan device	Processing software	Clinical relevance
Blanck-Lubarsch et al. [17]	Children with FAS	Mean age 8.8 years (Patients) Mean age 8.2 years (Controls)	15 male and 15 female (Patients) 18 male and 12 female (Controls)	3D measurement system based on the fringe projection technique developed at the University Hospital Muenster	Atos Professional V8 (Carl Zeiss GOM Metrology GmbH)	3D facial measurements provide objective metrics for FAS diagnosis, showing significantly shorter midface length and longer philtrum in FAS patients, which can serve as additional diagnostic criteria to current visual assessment methods.
Piedra-Cascón et al. [18]	Completely dentate participants	NR	2 males and 8 females	Face Camera Pro Bellus (Bellus3D Inc., Campbell, CA, US) with Huawei MediaPad M3 (Huawei, Shenzhen, China)	Face Camera App (Bellus3D Inc.)	The selected facial scanner provided a reliable method for digitizing a patient's extraoral soft tissues that could be considered when creating a virtual patient for treatment planning.
Revilla-León et al. [19]	Completely dentate participants	Over 18 years	20 laypersons 20 dental students 20 dentists	Bellus3D Face Camera PRO (Bellus3D Inc.) iTero Element (Align Technology, Redwood, CA, US) ScanBodyFace (AFT Dental System, Seville, Spain) ScanBodyMouth (AFT Dental System)	Dental Systems (3Shape, Copenhagen, Denmark) Matera 2.4 (exocad GmbH, Darmstadt, Germany)	3D and 2D simulations affect perception differently, with 3D receiving higher aesthetic ratings for the same discrepancies. Patient perceptions may differ when viewing their own dental features versus others'. Understanding these differences is crucial for effective treatment planning communication.
Revilla-León et al. [20]	A female model	NR	1 female	Bellus3D Face Camera PRO (Bellus3D Inc.) iTero Element (Align Technology, Redwood, CA, US) ScanBodyFace (AFT Dental System) ScanBodyMouth (AFT Dental System)	Dental Systems (3Shape) Matera 2.3 (exocad GmBH)	The esthetic perception of dental parameters may vary among dentists, dental students, and laypersons depending on the technological combination selected, namely 2D (photography) or 3D (facial scan) for superimposition with the digitized dentition.
Awuti et al. [21]	Patients with mandibulectomy	Mean age 67.25 years	9 male and 11 female	Vivid910 (Konica Sensing, Inc., Osaka, Japan)	Artec 3D Studio (Artec, Palo Alto, CA, US) Mimics11.11 3D modeling software (Materialise, Leuven, Belgium) GOM Inspect V8 (Carl Zeiss GOM Metrology GmbH)	Mandibular prostheses significantly improve facial asymmetry in segmental mandibulectomy patients. 3D evaluation provides objective evidence for prosthetic rehabilitation outcomes. Results can guide clinicians in prosthetic design and adjustment.
Danneels et al. [22]	Patients with WBS	Age of 6 years or older	8 male and 9 female (Patients) 15 male and 18 female (Controls)	VECTRA H1 system (Canfield Scientific, Parsippany, NJ, US)	Matlab2017b (Natick) VistaDent AT 3.1 software (GAC international, Bohemia, NY, US)	Early detection of WBS with 3D facial scanning allows for timely intervention and treatment planning. Detailed knowledge of craniofacial features helps optimize orthodontic treatment for WBS patients. These findings support efficient pre-screening prior to genetic testing.
Denadai et al. [23]	Skeletal Class III deformity, Anteromedial cheek deficiency	23.8 ± 5.4 years (Synchronous group) 24.2 ± 5.9 years (Isolated surgery group)	20 females (Synchronous group) 20 females (Isolated surgery group)	Used CBCT but no device information	Geomagic (3D Systems Corporation, Rock Hill, SC, US) 3dMDVultus (3dMD LLC, Atanta, GA, US) SimPlant O&O (Materialize)	Synchronous orthognathic surgery with fat grafting improves facial aesthetics and midface volume compared to isolated surgery alone, providing an effective solution for patients with anteromedial cheek deficiency.
Friscia et al. [24]	Patients with Class III undergoing orthognathic surgery	Mean age of 25.6 years (Hilotherm) Mean age of 24.1 years (Conventional Therapy) Mean age of 23 years (No Intervention)	15 males and 20 females (Hilotherm) 14 males and 18 females (Conventional Therapy) 3 males and 4 females (No Intervention)	Bellus3D Face Camera Pro System (Bellus3D Inc.) with iPhone (Apple Inc., Cupertino, CA, US)	Geomagic Design X 3D software (3D Systems Corporation)	Hilotherapy with controlled temperature shows superior effectiveness in reducing post-orthognathic facial edema compared to ice packs. The iPhone-based 3D scanning provides a practical solution for clinical assessment of facial swelling.
Horn et al. [25]	Volunteers	21–35 years	214 males and 399 females	3D stereophotogrammetry system (3dMD LLC)	VIEWBOX 4.1 (dHal Software, Kifissia, Greece)	Proportionally wider smiles are perceived as more attractive, particularly in females. This provides important diagnostic criteria for clinicians improving facial aesthetics. The findings can guide treatment planning in orthodontics and orthognathic surgery.
Pellitteri et al. [26]	Healthy subjects	25-48 years	11 males and 14 females	Face Hunter (Zirkonzahn GmbH) Bellus3D Dental Pro app with iPad Pro and iPhone X (Apple Inc.)	Geomagic X (3D Systems Corporation)	3D facial scanning provides a non-invasive alternative to traditional imaging for orthodontic diagnosis. Validation of the accuracy of these scanners supports their clinical implementation for facial analysis.
Blanck-Lubarsch et al. [27]	Children with FAS	Mean age 8.8 years (Patients) Mean age 8.2 years (Controls)	15 male and 15 female (Patients) 18 male and 12 female (Controls)	3D measurement system based on the fringe projection technique developed at the University Hospital Muenster	Python (version 3.7.1) Scikit-learn library (version 0.21.3)	The machine learning methods, particularly decision trees, demonstrated high accuracy (89.5%) in diagnosing FAS using only three facial measurements. This approach could provide clinicians with an objective, reliable, and easy-to-use diagnostic tool for FAS screening, potentially leading to earlier diagnosis and intervention.
Eliasova et al. [28]	Dentofacial irregularities of the jaws, Injury	Mean age 23.57 years	14 males and 18 females	CBCT KaVo X-ray and Cone Beam Unit (KaVo Dental GmbH, Biberach, Germany) Vectra 3dMD non-invasive, ambient light-based scanner (Canfield Scientific)	Mirror Photo Tools (Canfield Scientific)	The 3D comparison methods provide specific data on surgical outcomes using both CBCT and facial scanner technologies. The results show significant variations in facial morphology following maxillofacial surgery. These findings have applications in both surgical planning and forensic identification.
Lee et al. [29]	Skeletal III dentofacial deformity	At least 21 years	10 patients	Performed CBCT and 3D facial stereophotogrammetric scans but no device information	ProPlan CMF software (Materialise) Geomagic Freeform (3D Systems Corporation) 3Dvultus (3dMD LLC)	The accuracy of 3D soft tissue prediction is critical for surgical planning and patient communication, especially when considering ethnic differences. This study validates the reliability of ProPlan CMF for Chinese patients, showing clinically satisfactory results with mean absolute differences within 2 mm.
Raffone et al. [30]	Patients	NR	4 males and 6 females	Bellus3D Dental Pro-App (Bellus3D Inc.) with IPad Pro (Apple Inc.)	MeshLab (Visual Computing Lab, Pisa, Italy) GOM inspect (Carl Zeiss GOM Metrology GmbH)	Low-cost, handheld scanners provide clinically acceptable accuracy for integrating facial scanning into the digital dental workflow. The Slider Technique demonstrated better clinical usability with fewer scans required than the Free Technique. This technology provides an economical solution for integrating facial scanning into dental practices.
Revilla-León et al. [31]	Completely dentate participants	Over18 years	20 laypersons (9 males and 11 females) 20 dental students (10 males and 10 females) 20 dentists (6 males and 14 females)	Bellus3D Face Camera PRO (Bellus3D Inc.) iTero Element (Align Technology, Redwood, CA, US) ScanBodyFace (AFT Dental System) ScanBodyMouth (AFT Dental System)	Dental Systems (3Shape) Matera 2.4 (exocad GmBH)	Both 2D and 3D facial and intraoral digital scan superimpositions provided esthetically pleasing representations, which may be incorporated into private practice to visualize the treatment outcome.
Revilla-León et al. [32]	Complete maxillary dentate participants receiving a dental implant treatment in the mandible	Over18 years	10 patients	i-CAT FLX V-Series (KaVo Dental GmbH) Bellus3D Face Camera PRO (Bellus3D Inc.) with Huawei MediaPad M3 (Huawei)	Face Camera App (Bellus3D) Matera 2.4 (exocad GmBH)	The virtual patient, obtained by superimposing facial and intraoral scans guided by scan bodies, may facilitate 3-dimensional representation; however, diagnostic trial restorations are still recommended.
Xu et al. [33]	Mandibular prognathism, Mandibular deviation	Mean age of 21.2 ± 3.5 years	12 males and 18 females	Morpheus 3D (Morpheus, Gyoung-gi, Korea) CBCT scanner (KaVo Dental GmbH)	MDS software (Morpheus)	Isolated mandibular BSSRO surgery improves both upper and lower lip asymmetry. Transverse mandibular correction primarily influences lip symmetry improvement. Surgeons should be aware of philtrum lengthening as a potential side effect.
Zhu et al. [34]	Facial asymmetry with a mandibular deviation	18-35 years	-	Face Scan 3D sensor system (3D-Shape Corp., Erlangen, Germany)	Geomagic Studio 2013 (3D Systems Corporation)	The PWPA algorithm provides a more suitable and accurate SRP for 3D facial models of patients with mandibular deviation, achieving results comparable to expert assessment. This method improves the analysis of facial asymmetry and could enhance treatment planning in orthodontics and maxillofacial surgery.
Zupan et al. [35]	Patients with an transverse discrepancy between the maxilla and mandible	Median age of 26.0 ± 9.0 years	7 males and 8 females	Artec MHT (Artec 3D, Luxembourg, Luxembourg)	RapidForm 2006 (INUS Technology, Seoul, Korea)	SARME not only widens the maxillary arch but also affects overall facial aesthetics through soft tissue changes. Understanding these changes helps in treatment planning and patient communication, especially for patients with combined sagittal and transverse deficiencies.
Aljawad et al. [36]	NR	Mean age 20.5±9.2 years	14 males and 11 females	CBCT scan and a 3D facial scan ARC-1 facial scanner (Bellus3D Inc., Campbell, CA, US)	Bellus3D scanner application (Bellus3D Inc) Invivo5 software (version 5.4; Anatomage, Santa Clara, CA, US)	Low-cost facial scanners may provide an accessible alternative for clinical facial assessment. The integration with CBCT enables better evaluation of soft tissue and treatment planning. These scanners offer advantages in being inexpensive, portable, and easy to use in daily practice.
Andrews et al. [37]	Healthy participants	25–50 years	6 males and 23 females	3dMDface System (3dMD LLC, Atanta, GA, US) Bellus3D Face app (Bellus3D Inc.) with iPhone 11 Pro (Apple Inc.)	Geomagic Control X (3D Systems Corporation)	The iPhone TrueDepth NIR camera with Bellus3D app provides a portable, low-cost alternative for 3D facial imaging. This system demonstrates clinically acceptable accuracy for routine facial analysis and treatment planning.
Cascos et al. [38]	Healthy participants	Mean age 26.4 years	13 males and 47 females	Bellus 3D Face Camera PRO (structured light facial scanner)	MeshLab (Visual Computing Lab)	3D facial scanning accuracy is essential for diagnosis and treatment planning in prosthodontics, orthodontics, and surgery. This study validates the clinical reliability of dual-structured light scanning over traditional 2D photography for facial analysis.
Hou et al. [39]	Skeletal class III malocclusion	Mean age of 26.7 years	21 males and 37 females	NewTom Scanner (NewTom AG, Marburg, Germany) 3dMDTrio System multicamera (3dMD LLC)	ProPlan CMF software (Materialize) Geomagic Studio 2013 software (3D Systems Corporation)	The accuracy of soft tissue prediction can be more clearly analyzed by maxillofacial regional aesthetic units, giving clinicians a deeper understanding of how to use the software to predict soft tissue changes after orthognathic surgery.
Nogueira et al. [40]	Healthy volunteers	Over 18 years	5 males and 6 females	Cloner scanning cabin (dOne 3D, S˜aoPaulo, Brazil)	CloudCompare v.2.6.1 (CloudCompare)	Scanners with accurate 3D model reproductions and reliable digital measurements provide more precise diagnosis and better planning in orofacial treatment, while offering the advantages of data storage and longitudinal patient monitoring without radiation exposure.
Doan et al. [41]	NR	29–34 years	1 male and 3 females	Bellus3D ARC-7 (Bellus3D Inc.)	3D Builder (Microsoft, Seattle, WA, US)	The Bellus3D ARC-7 system provides accurate and reproducible 3D facial scans that can potentially reduce the need for radiographic exposure in orthodontic diagnosis and treatment planning, offering a non-invasive alternative for comprehensive facial analysis.
Ercal et al. [42]	Patients in osteoporosis after single dental implant placement	Mean age 66.2 ± 6.72	11 females	3dMDface System or 3dMDbody System (3dMD LLC)	3dMDVultus (3dMD LLC)	The use of facial scanning technology provides a noninvasive method to monitor post-implant tissue changes. This can help predict and assess postoperative swelling and complications. The technology may lead to more personalized surgical treatment approaches.
Farronato et al. [43]	skeletal class I	Mean age 35.97 years	NR	Planmeca ProMax 3D ProFace (Planmeca, Helsinki, Finland)	Mimics Innovation Suite 19 (Materialise) VAM (Vectra Analysis Module; Canfield Scientific) Romexis 6.4 (Planmeca)	Non-invasive 3D facial scanning may serve as an initial diagnostic tool in orthodontics, reducing radiation exposure. Strong correlations between soft and hard tissue measurements suggest facial scans can help predict skeletal structures.
Jearanai et al. [44]	Facial asymmetry with skeletal Class II and III malocclusion	Mean age of 24.4 ± 3.79 years	25 males and 27 females	Planmeca Viso® G7 (Planmeca)	Dolphin Imaging v11.9 (Dolphin Imaging System, Los Angeles, CA, US)	3D virtual head models improve diagnostic accuracy and treatment planning for facial asymmetry, enabling better communication between orthodontists and surgeons while revealing previously overlooked vertical discrepancies in both skeletal classes.

**Table 4:** Data acquisition method and patient information of selected case and technical reports Abbreviations: NR, not reported; 3D, three-dimensional; CBCT, cone-bean computed tomography; CAD, computer-aided design; CLP, cleft lip and palate; TMD, temporomandibular disorders

Author	Patient information	Age and sex	Facial scan device	Intra oral scanner	Processing software	Clinical relevance
Coachman et al. [45]	NR	NR	3D face app (Bellus3D Inc., Campbell, CA, US) with movile device	Used but no device information	DSDApp 3D (DSD, Madrid, Spain)	DSDApp 3D enables single appointment digital smile design on mobile devices, making the digital planning process more accessible and efficient for everyday clinical practice.
Granata et al. [46]	Maxillary edentulous	NR	Bellus3D (Bellus3D Inc.) CS9300 (Carestream Health Inc., Rochester, NY, US)	CS3600 (Carestream Inc.)	DDS-Pro (Dentalica Spa, Milano, Italy) exocad (exocad GmbH, Darmstadt, Germany)	The clinical relevance of this technique lies in its cost-effective digital workflow that integrates facial scanning, intraoral scanning, and CBCT data while maintaining compatibility with traditional methods, providing an efficient solution for complex prosthetic treatment planning.
Lo Russo et al. [47]	Mandibular edentulous	NR	Bellus3D FaceApp (Bellus3D Inc.)	TRIOS 3 color (3Shape, Copenhagen, Denmark)	3Shape Dental System software (3Shape) MeshLab (Visual Computing Lab, Pisa, Italy)	The integration of intraoral, perioral and facial scans into the digital denture design allows for a comprehensive 3D visualization of the tooth arrangement. This workflow requires only 5 minutes of additional time and eliminates the need for additional registration objects.
Park et al. [48]	Discolored composite restorations on maxillary anterior teeth with interdental diastemas	28-year-old, female	Bellus FacePro (Bellus3D Inc.) All in one system (AFT Dental System, Seville, Spain)	TRIOS 3 (3Shape)	Dental CAD Matera 2.4 (exocad GmBH)	The integration of facial scanning, intraoral scanning and additive manufacturing enables a precise digital workflow for direct composite restorations using a 3-piece silicone index system, offering improved efficiency and predictability compared to conventional methods.
Li et al. [49]	Fractured maxillary incisor	25-year-old, male	Bellus3D Dental Pro (Bellus3D Inc.)	CEREC AC (Dentsply Sirona, Charlotte, NC, US)	exocad dental CAD (exocad GmBH)	Virtual patient technology, which integrates digital scans and dynamic occlusion, provides predictable results for esthetic restorations, minimizing surgical errors and occlusal adjustments.
Pérez-Giugovaz et al. [50]	Partially edentulous, Periodontitis Stage II Grade A	NR	Bellus Face Camera Pro (Bellus3D Inc.)	CS3600 (Carestream Inc.)	Meshmixer (Autodesk, San Francisco, CA, US) Dental CAD Plovdiv (exocad GmBH)	This technology simplifies the fabrication of maxillary overdentures with an additively manufactured intraoral scan body that allows integration of digital impressions, occlusal registration and facial scans, streamlining the traditional prosthetic workflow.
Sun et al. [51]	Severe worn dentition	58-year-old, female	Face Hunter (Zirkonzahn GmbH, South Tyrolean, Italy)	TRIOS (3Shape)	Modellier v.6958 (Zirkonzahn GmbH)	This digital workflow integrates facial and intraoral scans to create a 3D virtual patient, enabling predictable prosthetic design with improved esthetics and functionality while minimizing chair-side adjustments.
Alisha et al. [52]	Cleft lip and palate	An infant	Bellus 3D facial scanner app (Bellus3D Inc.) with iPhone 11 Pro (Apple Inc., Cupertino, CA, US)	NR	MeshLab (Visual Computing Lab)	The frame enables quick and accurate 3D facial scans of infants with CLP without head movement, improving assessment of treatment outcomes and growth changes.
Beretta et al. [53]	NR	NR	Bellus3D ProFace application (version 1.6.11; Bellus3D Inc.) with iPhone 11 ProMax (Apple Inc.) Heges application (version 1.6.3; Marek Simonik) with iPhone 11 ProMax (Apple Inc.)	Used but no device information	Meshmixer (Autodesk)	Smartphone-based TrueDepth technology provides an accessible, portable and cost-effective alternative to traditional stereophotogrammetry for 3D pediatric orthodontic facial scanning, enabling frequent, radiation-free monitoring.
Campobasso et al. [54]	NR	25-year-old, female	EinScan H (Shining 3D Tech. Co. Ltd., Hangzhou, China)	TRIOS 3 color (3Shape)	Appliance Designer CAD software (3Shape)	This technique allows orthodontists to create a virtual patient for improved diagnosis and treatment planning through 3D digital smile design, analyze the patient's smile in the context of the lips, and move the teeth to achieve a balanced smile, all while avoiding unnecessary radiation exposure from CBCT scans.
Gupta et al. [55]	Frontal cranial defect	54-year-old, male	Bellus 3D ARC facial scanning software (Bellus3D Inc.) with mobile device	NR	Meshmixer (Autodesk)	Bellus 3D facial scanning with rapid prototyping offers an efficient digital workflow for cranioplasty fabrication, particularly beneficial for patients where conventional impressions are contraindicated due to site tenderness. The method enables precise prosthesis fabrication while reducing patient discomfort and treatment time.
Park et al. [56]	NR	NR	Used 3D facial scan and CBCT but no device information	Used but no device information	NR	Integration of CBCT, intraoral and facial scans creates accurate virtual patients, improving diagnosis and treatment planning while enhancing patient communication.
Yang et al. [57]	NR	NR	Face Hunter (Zirkonzahn GmbH)	iTero (Align Technology, Redwood, CA, US)	exocad (exocad GmbH) 123D Design (Autodesk)	This digital technique enables patient-specific virtual articulator mounting without conventional facebow procedures, streamlining the workflow while maintaining accuracy. It eliminates physical impressions and can be integrated with most virtual articulator systems.
Amin SA et al. [58]	Maxillary terminal dentition	A middle-aged male	Dental Pro (Bellus3D Inc.)	CEREC Prime Scan (Dentsply Sirona)	DentalCAD3 Galway (exocad GmbH)	The integration of 3D facial scanning with CBCT and intraoral scans provides a complete virtual patient representation, enabling more predictable esthetic and functional outcomes in full-arch implant rehabilitation while reducing the number of appointments and improving efficiency.
Garaicoa et al. [59]	Partially edentulous, Spacing between teeth, Generalized incisal and occlusal wear of all present dentition, Loss of vertical dimension of occlusion	48-year-old, female	Used 3D facial scan but no device information	iTero Element 5D (Align Technology)	DentalCAD 3.1 (exocad GmbH)	Integrating facial and intraoral scanners into digital dentistry enables predictable esthetic results and improved patient comfort. Digital workflows streamline treatment planning and delivery for full-mouth reconstructions.
Wang et al. [60]	Maxillofacial trauma	20-year-old, male	KaVo OP300-1 (KaVo Dental GmbH, Biberach, Germany) 3dMDface System (3dMD LLC, Atanta, GA, US)	NR	ProPlan CMF software (version 3.0; Materialize, Leuven, Belgium) Geomagic Wrap 2021 (3D Systems Corporation, Rock Hill, SC, US)	This digital workflow enables accurate prediction of facial soft tissue changes prior to prosthetic rehabilitation in maxillofacial trauma patients. The technique combines CBCT, 3D facial scanning and virtual planning to optimize prosthetic outcomes. This approach reduces chairside time and improves treatment predictability.
Yang et al. [61]	Patient requiring replacement of maxillary implant-supported fixed complete denture	A male	FaceSCAN3D Scientific Photolab 60 Hz (3D-Shape, Erlangen, Deutschland)	TRIOS (3Shape)	Geomagic Control X (3D Systems Corporation)	The blue screen approach with silicone matrix provides more accurate reproduction of the lip-tooth relationship in 3D facial scans, which is crucial for digital smile design and prosthetically driven implant planning, potentially leading to improved treatment outcomes.
Elbashti et al. [62]	Fully dentate	NR	Face Hunter (Zirkonzahn GmbH)	TORIOS 4 (3Shape)	GOM Inspect (Carl Zeiss GOM Metrology GmbH, Braunschweig, Germany) Meshmixer (Autodesk)	The protocol enables efficient creation of virtual dental patients by combining intraoral and facial scans using nasal geometry, eliminating the need for additional markers or CBCT while maintaining clinical accuracy.
Lee et al. [63]	TMD, Unstable occlusion with circular open bite, Severe skeletal Class II malocclusion	23-year-old, female	Used 3D facial scan and CBCT but no device information	Used but no device information	NR	The use of a virtual articulator and a digital mandibular position indicator enables accurate diagnosis and analysis of occlusal conditions in TMD patients with unstable occlusion, while the integration of digital technologies provides precision and efficiency in treatment.
Salloum [64]	NR	NR	VECTRA H2 facial scanner (Canfield Scientific, Parsippany, NJ, US)	CEREC Prime Scan (Dentsply Sirona)	exocad GmbH	This novel approach enhances the accuracy and efficiency of merging facial and intraoral scans in digital dentistry. The technique improves diagnostic precision and treatment planning through stable reference points. The method allows for better patient communication and predictable aesthetic outcomes in prosthetic dentistry.

The processing software documented in both tables showed significant variation, ranging from vendor-specific applications to general-purpose 3D processing software. Table 3 showed that clinical trials often used specialized analysis software for validation purposes, while Table 4 showed that case reports often used more practice-oriented software solutions focused on treatment planning and patient communication.

Clinical Applications and Implementation

The implementation and clinical applications documented in all four tables demonstrate a comprehensive integration of facial scanning technologies into dental practice. The patient information sections demonstrate successful application to diverse patient populations, with particular emphasis on the adaptability of the technology to different clinical needs. Clinical studies (Tables 1, 3) provided the scientific foundation by validating accuracy and standardizing scanning protocols. These studies validated mobile scanning solutions against traditional methods and demonstrated comparable accuracy for routine facial analysis.

Case reports and technical papers (Tables 2, 4) presented practical applications in various clinical scenarios, from digital smile design and orthognathic surgery planning to prosthetic rehabilitation. The clinical relevance sections in both Tables 3 and 4 showed how mobile solutions have increased accessibility by reducing costs and improving workflow integration while maintaining clinical accuracy.

The processing software sections documented successful integration with existing clinical platforms, demonstrating the technology's ability to complement established digital workflows. This integration was particularly evident in case reports (Table 4) where facial scan data was combined with other digital dental records for comprehensive treatment planning.

Discussion

Clinical Applications Across Dental Specialties

This scoping review provides a comprehensive analysis of the current state of facial scanning in clinical dentistry and identifies several key trends and implications for clinical practice. Our analysis of clinical applications and related research reveals significant differences in the use of facial scanning across dental specialties. Orthodontics and oral surgery show the most established implementation patterns, particularly in treatment planning and outcome evaluation [17,22-24,27,29,33-35,39,43,44,52-55,63]. In prosthodontics, facial scanning is increasingly integrated into digital workflows, particularly for smile design and complex rehabilitation cases [19-23,45-47,50,51,55,57-62,64]. Our review also identifies several specialties where facial scanning technology shows promising potential for future applications. For example, while applications in pediatric dentistry remain limited, our review identified several technical reports involving pediatric patients [52,53] and studies using facial scanning to diagnose conditions such as fetal alcohol syndrome [17,27] and Williams-Beuren syndrome [22], and cleft lip and palate [52]. Although these conditions may require multi-specialty care, facial scanning solutions could provide fast scanning speeds and improve comfort for pediatric patients [65].

Integration Into Digital Workflows

The integration of facial scanning into existing digital workflows emerges as a critical consideration across specialties. Although our review shows successful examples of integration in treatment planning and outcome evaluation, the lack of standardized protocols for combining facial scans with other digital dental records (such as CBCT and intraoral scans) remains a challenge. The need for established guidelines and standardized approaches to data integration will become increasingly important as artificial intelligence (AI) and automated analysis tools become more prevalent in dental practice [66,67].

Advances in Mobile-Based Facial Scanning Technologies

Another finding is the rapid transition to mobile device-based scanning solutions, particularly the Bellus3D system, as evidenced by both clinical studies and case reports. This shift means that facial scanning technology is becoming more accessible in dental practice. Although traditional professional systems remain important for specific applications, according to a meta-analysis by Mai and Lee [14], mobile solutions can achieve clinically acceptable accuracy with mean discrepancies ranging from 0.34 to 1.40 mm, which is within the established clinical threshold of <1.5 mm, despite lower overall accuracy compared to professional systems (standardized mean difference 3.96 mm, 95% confidence interval 2.81-5.10 mm) [14]. These mobile solutions offer advantages in terms of cost, accessibility and workflow integration. This development has the potential to significantly increase the adoption of facial scanning in routine dental practice.

The democratization of facial scanning technology through mobile solutions is particularly relevant when considering the geographic distribution of recent research. Our review of publications from 2020 to 2024 shows that current research activities are concentrated in specific regions, particularly the United States, China, and Italy. Although the presence of studies from 19 countries during this recent period indicates growing international involvement, the accessibility and cost-effectiveness of mobile solutions could play a critical role in expanding both research and clinical implementation in underrepresented regions. This technological shift can help ensure that the benefits of facial scanning are realized globally, particularly in regions where investment in traditional professional systems may be cost prohibitive.

Challenges and Limitations

In examining the current state of facial scanning implementation, several key challenges warrant attention. Although technical validation and accuracy studies are abundant, research on long-term clinical outcomes and treatment effectiveness remains limited. Integrating facial scanning into standard clinical workflows presents additional challenges, particularly in terms of data management and interoperability between disparate digital systems. In addition, as mobile solutions become more prevalent, issues of data security, patient privacy, and ethical considerations must be carefully addressed.

Future Research Directions

Future research directions should focus on addressing these identified gaps. Extensive validation of clinical outcomes through longitudinal studies is essential to establish evidence-based protocols. The development of standardized workflows, particularly for specialties where facial scanning is emerging, such as pediatric dentistry, requires systematic investigation. Although mobile scanning solutions offer promising opportunities and their adoption is steadily increasing, their trueness and precision still lag behind professional scanning systems [10,14]. Therefore, research to establish acceptable error thresholds for different dental specialties and/or cases would provide critical guidance to practitioners in choosing between mobile devices and professional scanners based on their specific clinical needs. Research on automated data integration systems and AI applications could improve the efficiency and effectiveness of facial scanning in clinical practice. In addition, studies examining barriers to implementation and cost-effectiveness in different clinical settings would provide valuable guidance for technology adoption.

## Conclusions

This scoping review shows that 3D facial scanning has been widely adopted in various dental specialties, from diagnosis and treatment planning to postoperative evaluation. Recent studies have confirmed that mobile device-based scanning, while slightly less accurate than dedicated systems, reduces costs and facilitates integration into digital workflows. However, gaps remain in standardizing scanning protocols and establishing long-term clinical evidence. Further research to validate these technologies in diverse patient populations, propose clear guidelines for data integration, and evaluate cost-effectiveness is essential to fully implement 3D facial scanning into routine practice.
